# Flavonoid-Rich Metabolite Profiling and Anti-Inflammatory Activity of *Adonis aestivalis* L.

**DOI:** 10.3390/molecules31162858

**Published:** 2026-08-16

**Authors:** Saule Orynbekova, Wirginia Kukula-Koch, Zuriyadda Sakipova, Zoya Allambergenova, Gulnara Kadyrbayeva, Bakhtiyar Minbayev, Bashaer Alsharif, Fabio Boylan

**Affiliations:** 1 School of Pharmacy, S.D. Asfendiyarov Kazakh National Medical University, Almaty 050000, Kazakhstan; saule_04_94@mail.ru (S.O.); sakipova.z@kaznmu.kz (Z.S.); chilnara_k@mail.ru (G.K.); 2 Department of Pharmacognosy with Medicinal Plants Garden, Medical University of Lublin, 1 Chodzki Street, 20-093 Lublin, Poland; 3 Scientific and Practical Control and Analytical Laboratory of Chemistry and Pharmacognosy, S.D. Asfendiyarov Kazakh National Medical University, Almaty 050000, Kazakhstan; bominbayev@mail.ru; 4 Faculty of Pharmacy, Umm Al-Qura University, Makkah 21955, Saudi Arabia; alsharib@tcd.ie; 5 School of Pharmacy and Pharmaceutical Sciences, Panoz Institute, and Trinity Biomedical Sciences Institute, Trinity College Dublin, Dublin 2, D02 PN40 Dublin, Ireland; fabio.boylan@tcd.ie

**Keywords:** *Adonis aestivalis*, flavonoids, HPLC–MS, anti-inflammatory activity, RAW 264.7, cytokines

## Abstract

*Adonis aestivalis* L. is a member of the genus *Adonis*, traditionally associated with cardiotonic compounds; however, its polyphenolic composition and anti-inflammatory potential warrant further phytochemical and pharmacological evaluation. The aim of this study was to characterise the metabolic profile of *A. aestivalis* extracts, with a focus on flavonoid-containing components, and to evaluate their anti-inflammatory activity in LPS-stimulated RAW 264.7 macrophages. Aqueous, 50% ethanolic, and absolute ethanolic extracts were analysed by HPLC–ESI–QTOF-MS/MS in positive and negative ionisation modes. The ethanolic extracts exhibited the most diverse chromatographic profiles, with a predominance of flavonoids, including derivatives of kaempferol, quercetin, and apigenin, as well as C-glycosylated flavones such as orientin, isoorientin, vitexin, and isovitexin. Phenolic acids, fatty acid derivatives, organic acids, and adonitol were also tentatively identified. The occurrence of glycosylated, acylated, and glucuronidated flavonoid derivatives indicates marked structural diversity within the flavonoid fraction. In biological assays, the 50% ethanolic extract showed no cytotoxicity towards RAW 264.7 cells at concentrations up to 50 μg/mL and produced a statistically significant but moderate reduction in LPS-induced NO production. Furthermore, it attenuated the release of pro-inflammatory cytokines, including IL-6, IL-1β, and TNF-α, to varying degrees. The data obtained indicate that *A. aestivalis* is a promising source of flavonoid-containing extracts with anti-inflammatory potential and may provide a foundation for further studies aimed at developing standardised phytochemical preparations with a defined polyphenolic profile. Further quantitative phytochemical analysis, bioactivity-guided fractionation, mechanistic studies, safety assessment, and in vivo evaluation are required to confirm the active constituents and therapeutic relevance of the extracts.

## 1. Introduction

Medicinal plants remain an important source of structurally diverse bioactive compounds with significant pharmacological potential. Among them, members of the genus *Adonis* L. in the family *Ranunculaceae* are of particular interest as plants containing cardiotonic compounds, primarily cardiac glycosides and cardenolides. It has previously been demonstrated that *Adonis aestivalis* L. contains cytotoxic cardiac glycosides, confirming the pharmacological significance of this species and explaining the traditional interest in the cardenolide profiles of plants belonging to the genus *Adonis* [1]. However, studying cardiac glycosides alone does not fully reveal the chemical and biological potential of these plants, since other groups of secondary metabolites, particularly polyphenolic compounds, may also contribute significantly to the biological activity of their extracts.

In recent years, research into the flavonoid profiles of species belonging to the genus *Adonis* has become increasingly important. Flavonoids are of interest not only as biologically active compounds but also as compounds of chemotaxonomic significance. Thus, in a study of the *Adonis amurensis* complex using flowers and leaves, 19 flavonoid compounds were isolated and identified, including C-glycosylflavones, O-glycosylflavones, and O-glycosides of flavonols. *A. amurensis* was characterised by a greater diversity of C-glycosylflavones than closely related taxa [2]. Furthermore, studies of *A. paryadrica* and *A. ramosa* demonstrated that the contents of phenolic compounds and flavonoids were associated with the antioxidant, enzyme-inhibitory, antimicrobial, and other biological activities of their extracts [3,4]. These findings confirm that polyphenolic compounds represent an important, yet still incompletely characterised, component of the phytochemical profile of the genus *Adonis*.

Although several *Adonis* species have been investigated with respect to their flavonoid composition, *A. aestivalis* remains insufficiently characterised in terms of its polyphenolic metabolome. Previous studies of this species have focused predominantly on cardiac glycosides, whereas comprehensive high-resolution profiling of flavonoids and related phenolic compounds remains limited. Consequently, the chemical diversity of these constituents and their potential biological relevance are still insufficiently understood. Comprehensive HPLC–ESI–QTOF-MS/MS characterisation of the metabolite profile of *A. aestivalis* therefore represents an important step towards expanding current phytochemical knowledge of this medicinal species and enabling the tentative annotation of metabolites that may not have been previously reported in *A. aestivalis*.

The pharmacological significance of flavonoids is particularly relevant in the study of plants containing cardiac glycosides. These natural polyphenols possess antioxidant, anti-inflammatory, anti-atherogenic, antithrombotic, and cardioprotective properties, and their effects may involve the regulation of various molecular targets and signalling pathways, including NF-κB, MAPK/ERK/JNK/p38, PI3K–Akt–eNOS, STAT3, and AMPK–mTOR [5]. In addition, certain flavonoids may influence the pharmacokinetics of cardiotonic compounds. Zhang and Morris demonstrated that biochanin A and silymarin inhibited P-glycoprotein-mediated efflux in Caco-2 cells, increased the intracellular accumulation of digoxin, and altered its bidirectional transport across the cell monolayer [6]. Consequently, flavonoids may be regarded not only as secondary components of plant materials but also as biologically active compounds capable of contributing to the overall effects of plant extracts.

Recent experimental studies have further emphasised the phytochemical diversity of the genus *Adonis*. Comparative GC–MS analysis of nine *Adonis* species revealed substantial interspecific differences in their chemical profiles and antioxidant, antimicrobial, and antiproliferative activities [7]. An organ-specific study of *A. paryadrica* also demonstrated differences in phenolic and flavonoid contents and biological activities among extracts obtained from the leaves, flowers, and roots [8]. These findings highlight the importance of species-specific and plant-organ-specific phytochemical characterisation rather than extrapolating results obtained for one *Adonis* species to another.

Recent studies of individual flavonoids related to those expected in flavonoid-rich plant extracts also provide a relevant pharmacological context. Kaempferol-3-O-rutinoside was shown to attenuate inflammatory responses, including the production of pro-inflammatory cytokines and the expression of iNOS and COX-2, in LPS-stimulated RAW 264.7 macrophages [9]. Similarly, quercetin and rutin reduced NO production and COX-2 levels in the same cellular model [10]. These findings support further investigation of flavonoid-containing extracts in macrophage-based inflammatory models. However, biological effects reported for isolated flavonoids cannot be directly attributed to a chemically complex *A. aestivalis* extract unless the corresponding compounds are quantified, isolated, and tested individually.

Thus, a comprehensive study of the flavonoid profile of *A. aestivalis* represents a relevant area of research that complements traditional investigations of cardenolides. Despite the available data on cardiac glycosides in this species, its polyphenolic composition remains insufficiently characterised. The novelty of the present study lies not in the use of standardised screening methods themselves, but in the integrated high-resolution characterisation of the flavonoid-rich metabolite profile of the insufficiently investigated species *A. aestivalis* and the preliminary evaluation of the anti-inflammatory activity of its selected extract. Accordingly, this study aimed to characterise the metabolite profiles of aqueous, 50% ethanolic, and absolute ethanolic extracts of *A. aestivalis* using HPLC–ESI–QTOF-MS/MS, with particular emphasis on flavonoid-containing constituents, and to evaluate the anti-inflammatory activity of the selected 50% ethanolic extract in LPS-stimulated RAW 264.7 macrophages.

## 2. Results

### 2.1. Chemical Profiling of A. aestivalis Extracts

Considering the limited phytochemical information currently available for *A. aestivalis*, this analysis was undertaken to establish the first comprehensive metabolite profile of this species using high-resolution LC–ESI–QTOF-MS/MS. Comprehensive fingerprint profiling of aqueous, 50% ethanolic, and absolute ethanolic extracts was performed using HPLC–ESI–QTOF-MS/MS in both positive and negative ionisation modes. Comparative analysis of the base peak chromatograms (Figure S1, Supplementary Materials) revealed pronounced differences in metabolite distribution depending on the extraction solvent. In both ionisation modes, the ethanolic extracts exhibited a high number of resolved peaks, particularly in the mid- and late-retention time regions (approximately 18–25 min), indicating a greater abundance of moderately polar and semi-polar secondary metabolites. In contrast, the aqueous extract showed a dominance of early-eluting compounds (below 10–12 min), consistent with highly polar constituents, and a markedly lower signal intensity in the flavonoid-rich retention window. The 50% ethanolic extract displayed an intermediate profile, combining both early polar metabolites with a moderate representation of flavonoid-derived peaks. Overall, the chromatographic fingerprints demonstrate that 50% ethanol was efficient for extracting structurally diverse secondary metabolites from the studied *Adonis* material. All fingerprints are presented in Figure S1, whereas the tentative compositional assignment of the ethanolic extract is summarised in Table 1.

The results of HPLC–ESI–QTOF-MS/MS analysis showed that *Adonis aestivalis* is characterised by a complex and diverse metabolic profile, predominantly composed of polyphenolic compounds. The identified metabolites included particularly noteworthy flavonoids, namely C-glycosylated flavones (orientin, isoorientin, vitexin, and isovitexin), glycosylated, glucuronidated and acylated derivatives of kaempferol and quercetin, but also phenolic acids, organic acids, and fatty acid derivatives, indicating an extensive diversification of secondary metabolism in this species. Flavonoids constituted the predominant class of compounds, as evidenced by their marked dominance in the chromatographic profiles. The detected components were characterised with good mass accuracy, with mass errors ranging from −7.86 to +6.69 ppm. In most cases, the mass error was below 5 ppm; however, for several low-mass compounds slightly larger deviations were observed. Therefore, accurate mass was interpreted together with MS/MS fragmentation patterns, retention behaviour, elemental composition, and comparison with published data to support the tentative metabolite assignments. For low-molecular-weight metabolites, accurate mass alone was considered insufficient for confident annotation and was interpreted together with characteristic MS/MS fragmentation patterns and literature data.

As the portfolio of flavonoids was rich, it was often difficult to unequivocally assign the compounds’ names; however the tentative identification was performed with the highest care. This was the case for kaempferol-*O*-diglucoside and orientin glucoside, which are isobaric compounds sharing the same elemental composition (C_27_H_30_O_16_). Their tentative assignment was based on the observed MS/MS fragmentation patterns rather than precursor mass alone. Nevertheless, unequivocal structural discrimination would require additional confirmation using authentic reference standards or NMR spectroscopy.

The obtained metabolic profile is consistent with previously published phytochemical data on the genus *Adonis*. For example, a review by Shang et al. showed that members of this genus are characterised by a wide range of secondary metabolites, including cardiac glycosides, flavones, carotenoids, coumarins, and other classes of compounds. In this regard, the predominance of flavonoids identified in the present study, including glycosylated and acylated derivatives of kaempferol, quercetin, and apigenin, confirms the phytochemical significance of *A. aestivalis* within the genus *Adonis*, whose members are characterised by the accumulation of flavones, flavonols, and their glycosylated derivatives, including kaempferol derivatives.

The MS/MS spectra of flavonoid compounds were interpreted taking into account the known fragmentation patterns of flavones, flavonols, and flavanones in negative ionisation mode. According to Fabre et al., negative-ion ESI-MS/MS is an informative tool for the structural characterisation of flavonoid aglycones, as it allows the identification of diagnostic fragment ions, RDA fragmentation, as well as characteristic neutral losses of CO, CO_2_, and C_3_O_2_ [11]. Therefore, the detection of diagnostic ions of the aglycones quercetin, kaempferol, and apigenin at *m*/*z* 301, 285, and 269, respectively, can be considered an important criterion for the preliminary identification of the corresponding flavonoid derivatives in the *A. aestivalis* extract.

The flavonoid fraction proved to be the most abundant and structurally diverse. In addition to kaempferol and its glycosylated derivatives, several C-glycosylated flavones were identified, including orientin, isoorientin, vitexin, and isovitexin, based on characteristic sugar ring cleavage fragments. The presence of both mono- and diglycosylated derivatives, as well as acylated forms, acetyl- and malonyl-substituted flavonoids, indicates intensive processes of glycosylation and post-glycosylation modification of the flavonoid core. Such structural diversity indicates active pathways of flavonoid modification in the plant.

The compound detected at *m*/*z* 609.1456 ([M–H]^−^) was tentatively identified as kaempferol-O-diglucoside. This identification is based on the exact mass and fragmentation pattern and is consistent with previously published phytochemical data on species of the genus *Adonis*, for which glycosylated derivatives of kaempferol have been described.

The preliminary identification of vitexin and isovitexin in the *A. aestivalis* extract is of particular interest, as these compounds belong to the C-glycosylated flavone class and can be considered analytically significant components of plant extracts. Thus, Yan et al. developed an LC–MS/MS method for the simultaneous determination of vitexin and isovitexin in rat plasma following oral administration of *Santalum album* L. leaf extract; for both compounds, the diagnostic mass transition *m*/*z* 431.2 → 311.1 was used, confirming the analytical significance of the *m*/*z* 311 fragment in the characterisation of vitexin- and isovitexin-like C-glycosides [12].

Furthermore, the preliminary identification of orientin-, isoorientin-, vitexin-, and isovitexin-like C-glycosylated flavones is consistent with the data from Zhang et al., who demonstrated the possibility of simultaneously determining vitexin, orientin, isoorientin, and their C-glycoside derivatives by LC–MS/MS using characteristic diagnostic mass transitions, including *m*/*z* 431 → 311 for vitexin and *m*/*z* 447 → 327 for orientin/isoorientin. Furthermore, the diagnostic fragment ions of orientin glucoside described in the work by Zhang et al. confirm the analytical significance of the fragments characteristic of orientin-like C-glycosides detected in the *A. aestivalis* extract [13]. It is worth noting that the tentative distinction between orientin and isoorientin, as well as vitexin and isovitexin, was based on chromatographic behaviour and comparison with literature data. However, these positional C-glycosyl isomers exhibit nearly identical MS/MS fragmentation in negative ionisation mode and cannot be unequivocally distinguished without authentic reference standards.

Complex quercetin conjugates were also identified, including mixed hexoside-glucuronide derivatives and their esterified forms. The presence of glucuronidated flavonoids is of particular interest, as such metabolites are less frequently reported in members of the Ranunculaceae family and may contribute significantly to the polarity and biological activity of the extract.

Another ion with *m*/*z* of 639.1203 ([M–H]^−^) was tentatively assigned as a quercetin-hexoside-glucuronide based on its characteristic fragmentation pattern. The MS/MS spectrum showed a prominent ion at *m*/*z* 463.0874, corresponding to the neutral loss of 176 Da, indicative of a glucuronic acid residue. Further fragmentation yielded a diagnostic ion at *m*/*z* 301.0349, consistent with the quercetin aglycone following subsequent loss of a hexose unit (162 Da). The observed fragmentation pathway (639 → 463 → 301) supports the presence of a mixed diglycoside composed of a hexosyl and a glucuronosyl moiety.

Another flavonoid detected at *m*/*z* 831.1615 ([M–H]^−^) was assigned the molecular formula C_37_H_36_O_22_ (DBE = 20), suggesting a highly oxygenated flavonoid conjugate. The MS/MS spectrum exhibited a coherent sequential fragmentation pathway. The precursor ion first generated a fragment at *m*/*z* 639.1200, corresponding to a neutral loss of 192 Da, followed by a fragment at *m*/*z* 463.0872 after the loss of a glucuronyl residue (176 Da). Further fragmentation yielded the diagnostic quercetin aglycone ion at *m*/*z* 301.0330 following the loss of a hexosyl moiety (162 Da). Thus, the fragmentation sequence 831.1615 → 639.1200 → 463.0872 → 301.0330 provides strong evidence for the presence of a quercetin-hexoside-glucuronide core carrying an additional esterifying substituent. The relationship between the ions at *m*/*z* 831.1615 and 639.1200 further supports this assignment, as the larger conjugate retains the same quercetin-hexoside-glucuronide fragmentation core observed for the ion at *m*/*z* 639.1203. Based on the accurate mass and fragmentation pattern, the compound was tentatively classified as an esterified quercetin conjugate. However, the exact identity, position, and nature of the additional esterifying substituent cannot be established solely from the available HRMS/MS data and require further structural confirmation.

A compound detected at *m*/*z* 593.1506 ([M−H]^−^) was tentatively identified as saponarin (isovitexin-7-O-glucoside) based on its characteristic MS/MS fragmentation pattern. The MS/MS spectrum showed a prominent fragment ion at *m*/*z* 431.0984, corresponding to the neutral loss of 162 Da and indicating cleavage of the O-linked hexose residue, yielding the isovitexin aglycone. Further fragmentation produced characteristic cross-ring cleavage ions at *m*/*z* 473.1086 and 311.0562, supporting the presence of the C-glycosyl flavone skeleton. This fragmentation pattern is consistent with the tentative assignment of the metabolite as saponarin. Although a low-abundance ion corresponding to the apigenin aglycone (*m*/*z* 269) was observed under higher collision energies, fragmentation of C-glycosyl flavones is predominantly governed by cross-ring cleavages rather than complete sugar loss, distinguishing them from O-glycosyl flavonoids. Therefore, the observed fragmentation pattern is consistent with the tentative identification of the metabolite as saponarin.

In addition to these compounds, another kaempferol derivative was tentatively identified. A compound with the molecular formula C_24_H_22_O_14_ (DBE = 14) was detected and exhibited a fragmentation pattern characteristic of an acylated kaempferol glycoside. The MS/MS spectrum showed a diagnostic fragment ion at *m*/*z* 285.0403 consistent with a kaempferol-type flavonoid, although *m*/*z* 285 alone is not sufficient to distinguish kaempferol from other isobaric flavonoid aglycones. Additional fragments at *m*/*z* 489.1057 and 429.0843 suggest the sequential loss of acyl and sugar-related moieties, while the presence of ions at *m*/*z* 357.0624 and 327.0527 is consistent with partially cleaved glycosidic residues. The observed fragmentation behaviour, together with the elemental composition, supports the tentative assignment of this metabolite as a malonylated kaempferol glucoside, likely a kaempferol-O-(malonyl)-hexoside derivative. However, the exact position of acylation and glycosylation cannot be conclusively determined based solely on MS/MS data.

Also, apigenin finds its place among the flavonoid aglycones present in the extracts. Its derivative with the molecular formula C_24_H_22_O_13_ (DBE = 14) was detected at *m*/*z* 517.0953 ([M–H]^−^) and tentatively assigned as an apigenin-O-malonyl-hexoside. The MS/MS spectrum exhibited a characteristic neutral loss of 44 Da (*m*/*z* 473.1026), corresponding to CO_2_ elimination, which is diagnostic for malonylated flavonoid glycosides. At higher collision energy, a prominent fragment at *m*/*z* 269.0370 was observed, consistent with the deprotonated apigenin aglycone, confirming the flavone core. Additional fragment ions at *m*/*z* 413.0796, 341.0590, and 311.0486 further supported sequential cleavage of acyl and glycosidic moieties. Based on the accurate mass and fragmentation behaviour, the metabolite was tentatively identified as an apigenin-O-(malonyl)-hexoside.

It is important to note that, due to the absence of full structural confirmation (e.g., NMR data), the assignments presented above should be regarded as provisional.

Phenolic acids were represented by chlorogenic acid and dicaffeoylquinic acid, identified through diagnostic quinic acid fragments (*m*/*z* 191) and characteristic caffeoyl-derived ions. These compounds, together with flavonoids, form a coherent phenylpropanoid-derived metabolic network.

The non-phenolic fraction comprised hydroxylated fatty acids and oxylipin-type metabolites, including dihydroxy-octadecanoic acid and conjugated linoleic acid derivatives. Their fragmentation patterns and relatively high retention times are consistent with lipid-derived structures, reflecting the presence of oxidised fatty acid metabolites within the extract.

The compound detected at *m*/*z* 151.0612 ([M–H]^−^) was preliminarily identified as adonitol with the molecular formula C_5_H_12_O_5_. The early retention time of 2.065 min indicates its high polarity, which is characteristic of sugar alcohols. In the MS/MS spectrum, fragment ions at *m*/*z* 119.0336, 101.0230, 89.0234, and 71.0130 were observed, associated with sequential dehydration and cleavage of the polyol carbon chain. The DBE value of 0 further confirms the saturated aliphatic nature of the compound.

Overall, the metabolite profile of *A. aestivalis* reveals an unexpectedly high diversity of secondary metabolites, comprising C-glycosylated flavones, flavonol glycosides, glucuronidated and acylated flavonoids, phenolic acids, fatty acid derivatives, and polyols. The coexistence of these chemically distinct metabolite classes highlights the metabolic complexity of this insufficiently investigated species and considerably expands the currently available phytochemical knowledge of the genus *Adonis*.

Based on the comparative phytochemical profiling of the aqueous, 50% ethanolic, and absolute ethanolic extracts, the 50% ethanolic extract was selected for subsequent biological evaluation. Compared with the aqueous and absolute ethanolic extracts, it exhibited an intermediate chromatographic profile, combining early polar metabolites with a moderate representation of flavonoid-derived peaks. This extract was therefore selected for the preliminary assessment of anti-inflammatory activity in the present study.

### 2.2. The Bioactivity Studies

#### 2.2.1. Cell Viability Assay

**Figure 1:**
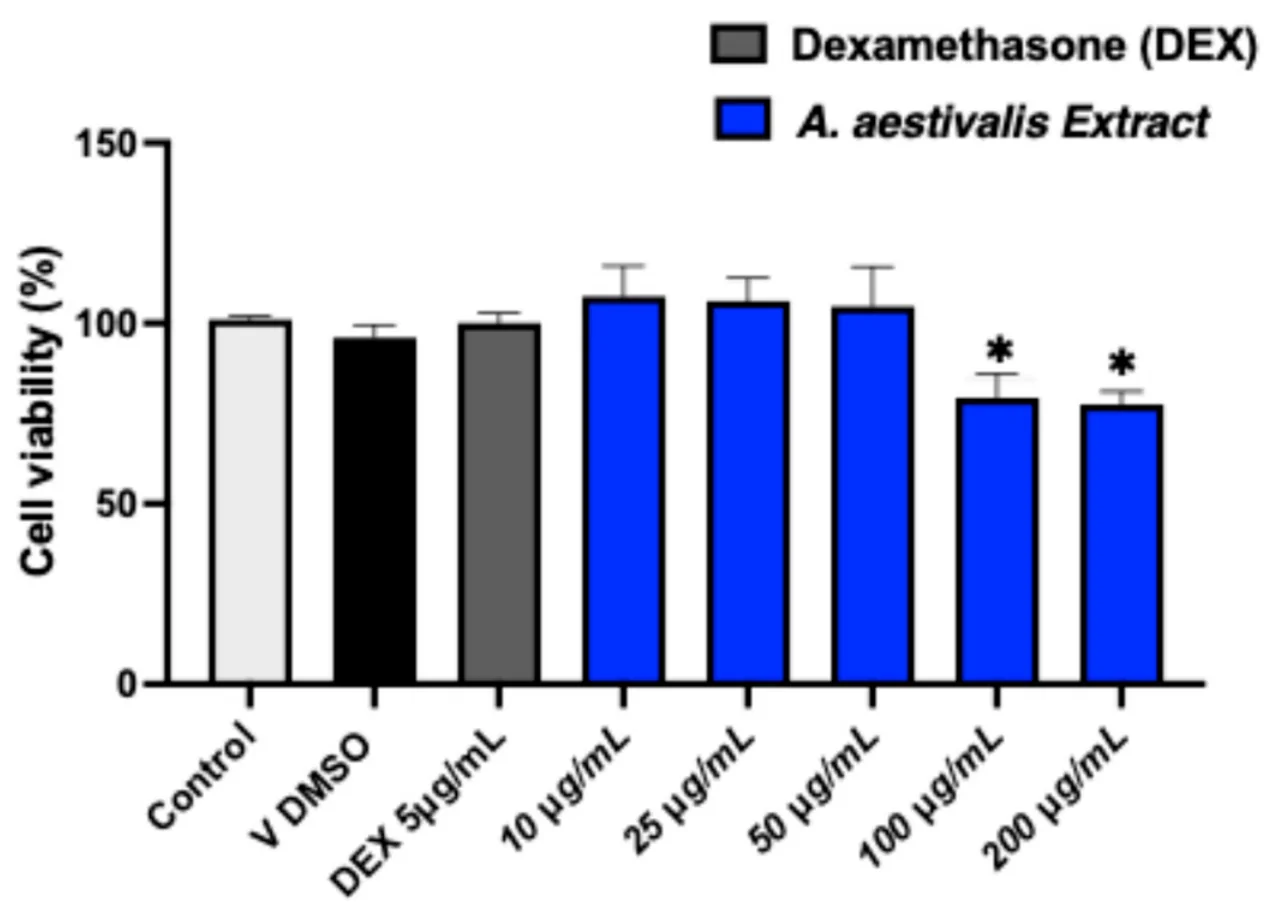
Effects of *A. aestivalis* extract on the viability of RAW 264.7 cells. The cells were treated with vehicle (DMSO), dexamethasone (DEX, 5 μg/mL), or *A. aestivalis* extract (10, 25, 50, 100, and 200 μg/mL) for 24 h. Cell viability was measured using the CCK-8 assay. Data are presented as the mean ± SD of at least three independent experiments. * *p* < 0.05 versus the control group.

The results obtained (Figure 1) showed no statistically significant differences in cell viability between the control group and the groups treated with *A. aestivalis* extract at concentrations of 10–50 μg/mL. *A. aestivalis* extract in the specified concentration range did not exert a cytotoxic effect on RAW 264.7 cells.

#### 2.2.2. Determination of NO Production

**Figure 2:**
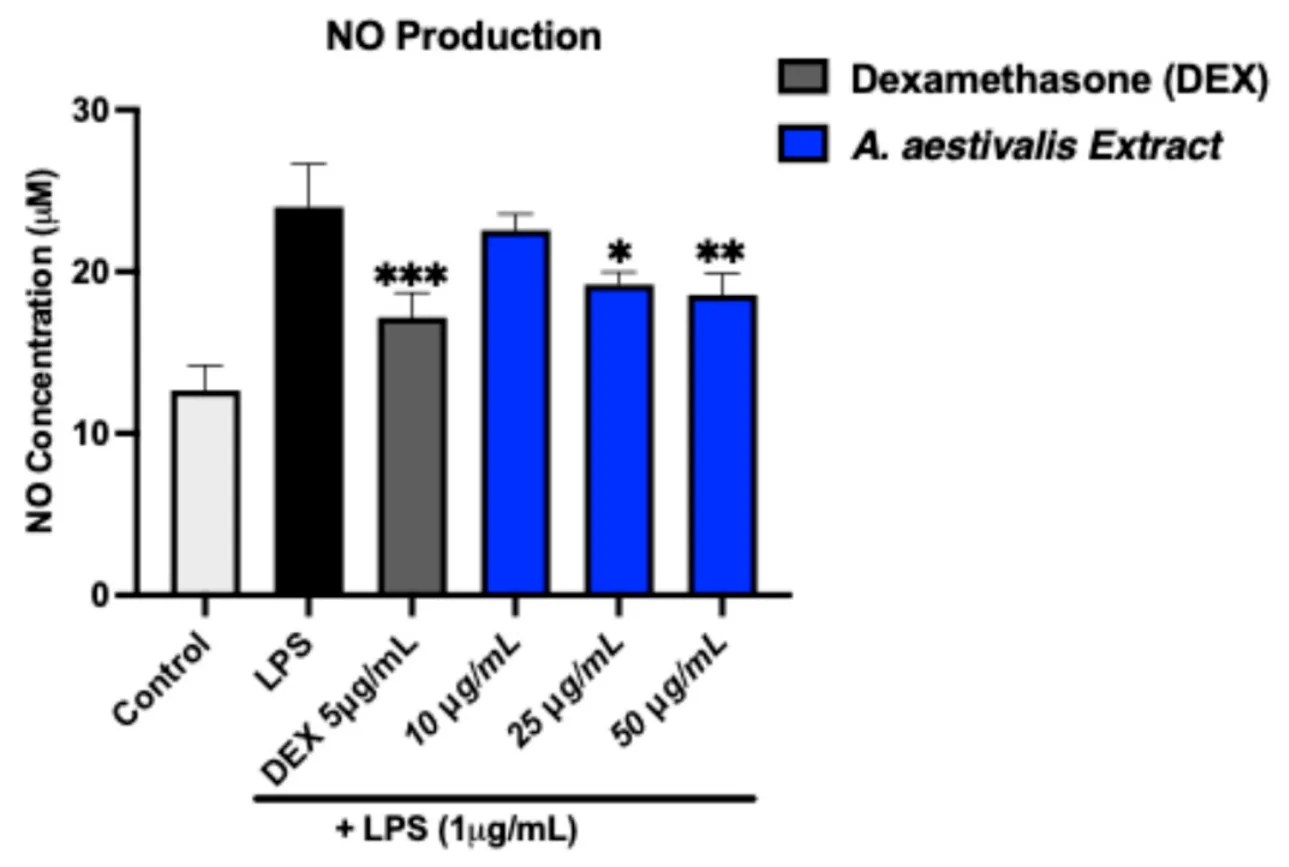
Effects of *A. aestivalis* extract on nitric oxide (NO) production in LPS-stimulated RAW 264.7 cells. RAW 264.7 cells were stimulated with LPS (1 μg/mL) and treated with dexamethasone (DEX, 5 μg/mL) or *A. aestivalis* extract (10, 25, and 50 μg/mL) for 24 h. Nitric oxide production was measured using the Griess assay. Data are presented as the mean ± SD of at least three independent experiments. * *p* < 0.05, ** *p* < 0.01, *** *p* < 0.001 versus the LPS group.

*A. aestivalis* extract reduced NO production in LPS-stimulated RAW 264.7 macrophages in a dose-dependent manner (Figure 2). Statistically significant suppression was observed at concentrations of 25 and 50 μg/mL (*p* < 0.05 and *p* < 0.01, respectively), suggesting a moderate anti-inflammatory effect under the experimental conditions.

#### 2.2.3. Determination of Cytokine Production in Cell Supernatant

**Figure 3:**
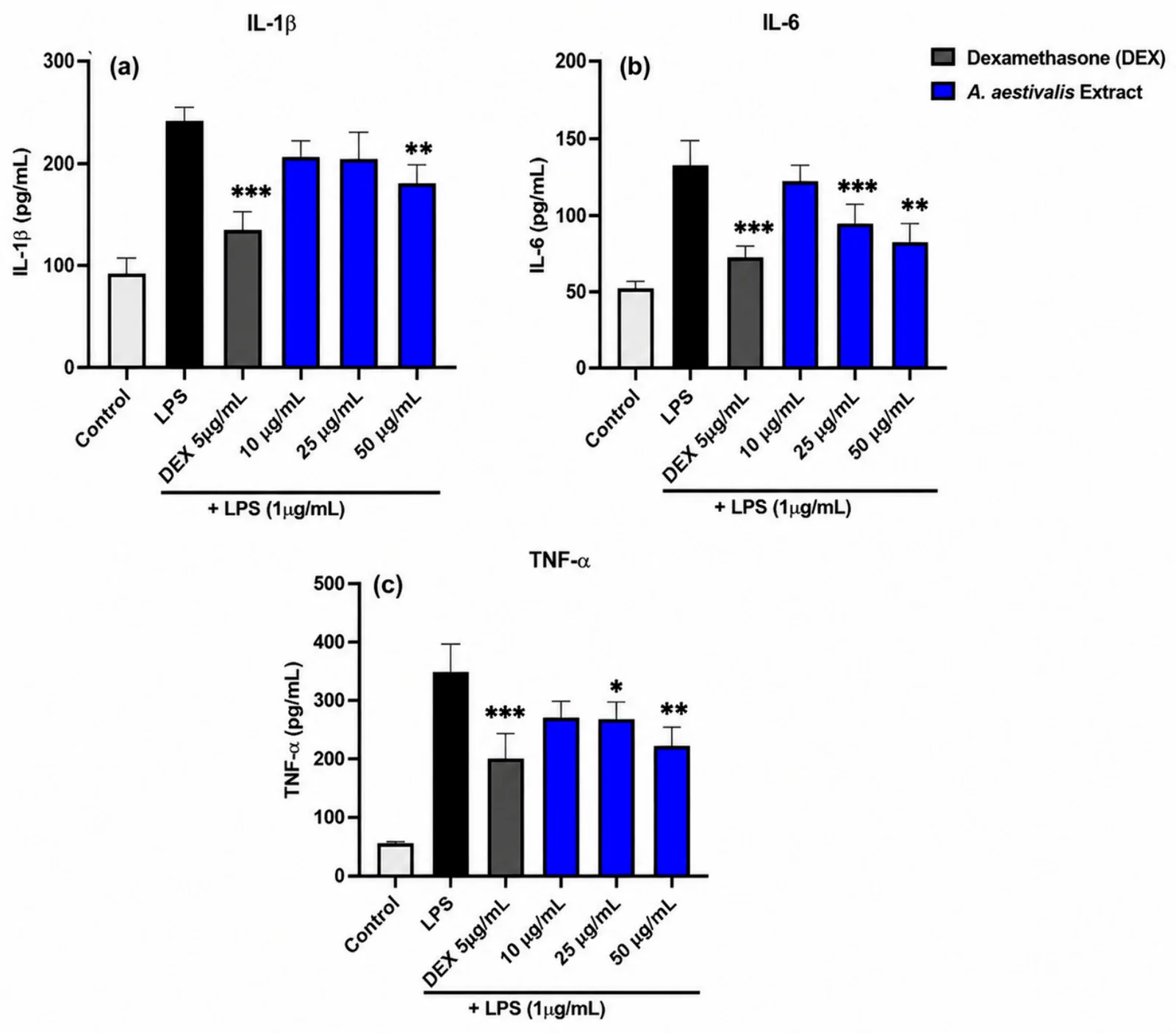
Effect of *A. aestivalis* extract on inflammation-related cytokines in RAW 264.7 cells. After treatment with LPS (1 µg/mL), DEX (5 µg/mL), or *A. aestivalis* extract (10, 25, and 50 µg/mL), cytokine production was measured using ELISA after 24 h. (**a**) IL-1β production; (**b**) IL-6 production; (**c**) TNF-α production. Data represent the mean ± SD of at least three independent experiments. * *p* < 0.05, ** *p* < 0.01, *** *p* < 0.001 versus the LPS group.

*A. aestivalis* extract reduced the production of the pro-inflammatory cytokines TNF-α, IL-6, and IL-1β in LPS-stimulated RAW264.7 cells (Figure 3). Significant reductions were observed, particularly at concentrations of 25 and 50 μg/mL, suggesting that the extract exerts anti-inflammatory effects under the experimental conditions.

## 3. Discussion

The metabolite profile obtained for *Adonis aestivalis* is generally consistent with the phytochemical characteristics previously reported for other members of the genus *Adonis*. According to a comprehensive review, more than 120 compounds have been described in this genus, including cardiac glycosides, flavonoids, carotenoids, coumarins, and other secondary metabolites. Flavonoids such as orientin, isoorientin, vitexin, luteolin, apigenin, kaempferol, and their glycosylated derivatives have been reported in *A. vernalis*, *A. coerulea*, *A. mongolica*, and *A. amurensis*. These findings support the important contribution of flavonoids, particularly glycosylated flavones and flavonols, to the phytochemical profile and possible chemotaxonomic differentiation of species belonging to this genus [14].

The present findings also demonstrate that the chemical composition of *Adonis* extracts may vary substantially among species, populations, and extraction conditions. Recent phytochemical evaluation of *A. volgensis* populations from Kazakhstan revealed variation in metabolite composition, biological activity, and toxicity, emphasising the importance of plant origin and extraction procedure when interpreting the pharmacological potential of *Adonis* preparations. Therefore, the metabolite profile reported for *A. aestivalis* should be regarded as characteristic of the investigated plant material and extraction conditions rather than as a universal profile of the species [15].

The simultaneous tentative detection of chlorogenic and dicaffeoylquinic acids together with oxidised C18 fatty acid derivatives indicates the presence of metabolites associated with phenylpropanoid metabolism and lipid oxidation. Chlorogenic acid and its derivatives are formed through the phenylpropanoid pathway, whereas oxidised fatty acid metabolites and oxylipins participate in plant defence, adaptation, and responses to environmental stress. However, the presence of these metabolites alone does not directly demonstrate the activation or interaction of the corresponding biosynthetic pathways in *A. aestivalis*. Confirmation of such pathway-level relationships would require targeted metabolomics, transcriptomic analysis, or measurements of relevant biosynthetic enzymes [16,17].

The tentative annotation of mixed glycosidic systems, including quercetin hexoside–glucuronide derivatives and an esterified quercetin conjugate, indicates extensive post-glycosylation modification within the flavonoid fraction. Flavonoid glucuronides and other highly substituted flavonoid conjugates have previously been described in plants belonging to the family Ranunculaceae. Glycosylation and glucuronidation increase molecular polarity and may affect the stability, transport, intracellular storage, and detoxification of flavonoids. These modifications are catalysed by UDP-dependent glycosyltransferases; for example, UGT84F9 has been identified as a major enzyme involved in the formation of flavonoid glucuronides, including quercetin derivatives, in Medicago truncatula. Nevertheless, the exact identities and substitution positions of the conjugates tentatively detected in the present study cannot be established from HRMS/MS data alone and require confirmation using authentic standards or NMR spectroscopy [18,19,20].

In addition to phenolic metabolites, adonitol, also known as ribitol, was tentatively annotated in the investigated extract. This highly polar pentahydric sugar alcohol has previously been reported in *A. vernalis*, *A. amurensis*, *A. annua*, and *A. microcarpa*. Its occurrence in *A. aestivalis* may therefore represent an additional chemotaxonomic feature of the genus and reflects the contribution of carbohydrate–polyol metabolism to the overall metabolite profile. Earlier pharmacognostic investigations also described adonite together with flavones and their C- and O-glycosides as characteristic constituents of *Adonis* species. However, the present study did not evaluate the biological contribution of adonitol, and no pharmacological effect should be attributed to this compound solely on the basis of its tentative detection [21,22,23,24].

Overall, the metabolite composition of *A. aestivalis* indicates considerable chemical diversity involving glycosylated, acylated, and glucuronidated flavonoids, phenylpropanoid acids, fatty acid derivatives, organic acids, and polyols. This profile complements previous information on the genus *Adonis* and expands current knowledge of the insufficiently investigated annual species *A. aestivalis*. At the same time, the analysis was qualitative and untargeted. Consequently, the absolute or relative concentrations of the tentatively annotated metabolites remain unknown, and the predominance of particular compounds cannot be established without validated quantitative analysis.

The biological results should be interpreted as preliminary in vitro evidence of anti-inflammatory potential rather than as confirmation of a therapeutic effect. The selected 50% ethanolic extract did not significantly reduce the viability of RAW 264.7 cells at concentrations up to 50 μg/mL. Therefore, the observed changes in inflammatory mediators at these concentrations were unlikely to result from nonspecific cytotoxicity. The extract produced a statistically significant but moderate reduction in LPS-induced NO production at concentrations of 25 and 50 μg/mL. Its effects on cytokine release varied among the measured mediators: IL-6 showed the most consistent reduction, whereas changes in IL-1β and TNF-α were more modest and occurred only at selected concentrations.

The reduction in NO production can be interpreted within the established biological role of nitric oxide in macrophage-mediated inflammation. Excessive NO formation in activated macrophages is commonly associated with inducible nitric oxide synthase. However, the present study measured only extracellular NO production and did not determine iNOS expression or enzymatic activity. Therefore, the results support attenuation of inflammatory activation but do not demonstrate direct inhibition of iNOS by the *A. aestivalis* extract [25].

The observed changes in IL-1β, IL-6, and TNF-α further support the anti-inflammatory potential of the extract because these cytokines are important mediators of macrophage inflammatory responses. Their expression is regulated through several interconnected signalling networks, including NF-κB and MAPK pathways. Nevertheless, NF-κB nuclear translocation, phosphorylation of MAPKs, and expression of downstream inflammatory genes were not examined in the present study. Consequently, involvement of NF-κB, MAPK, or related pathways should be presented only as a mechanistic hypothesis requiring experimental confirmation [26].

The flavonoid-rich composition of the extract provides a biologically plausible context for the observed activity. Flavonoids have been widely associated with modulation of oxidative stress, nitric oxide production, cytokine release, and intracellular inflammatory signalling. Therefore, the moderate inhibition of inflammatory mediators observed in the present study may reflect the combined contribution of several polyphenolic constituents. However, the detected kaempferol, quercetin, apigenin, orientin, isoorientin, vitexin, and isovitexin derivatives were not quantitatively determined, isolated, or tested individually. The activity cannot therefore be attributed to a specific flavonoid, and possible additive, synergistic, or antagonistic interactions among extract constituents remain unknown [27].

The present findings extend the group’s previous investigation of the perennial species *A. tianschanica*. In that study, the 50% hydroethanolic extract and isolated isoquercitrin produced a stronger reduction in LPS-induced NO, IL-6, TNF-α, and IL-1β production in RAW 264.7 macrophages. In comparison, the anti-inflammatory effect of the *A. aestivalis* extract was more moderate. This difference should not be attributed solely to the annual or perennial life form of the plants because that hypothesis was not directly tested. More plausible explanations include species-specific chemical composition, differences in the concentrations of active constituents, geographical origin, extraction conditions, and interactions among metabolites [28].

Anti-inflammatory activity has also been reported for a methanolic extract of the aerial parts of *A. coerulea*. That study identified a chemically diverse profile containing flavonoids and lipid-related metabolites and proposed PPAR-γ-mediated regulation of NF-κB as a possible mechanism of activity. Linolenic acid and licochalcone A were reported among the compounds associated with inhibition of LPS-induced NO production. These findings indicate that anti-inflammatory activity may occur in several *Adonis* species and may involve both polyphenolic and lipid-derived constituents. Nevertheless, the mechanism described for *A. coerulea* cannot be directly extrapolated to *A. aestivalis*, because PPAR-γ and NF-κB were not measured in the present investigation [29].

Several limitations should be considered. First, the metabolite assignments were tentative and were not confirmed using authentic reference standards or NMR spectroscopy. Second, no quantitative determination of the proposed metabolites was performed, and a validated chemical marker assay was not developed. Third, only one selected extract and one immortalised murine macrophage cell line were used for biological evaluation. Fourth, no bioactivity-guided fractionation or testing of isolated constituents was conducted. Fifth, molecular targets such as iNOS, COX-2, NF-κB, MAPKs, and PPAR-γ were not examined. Finally, the study did not evaluate bioavailability, pharmacokinetics, in vivo efficacy, repeated-dose toxicity, or possible cardiac effects.

**Figure 4:**
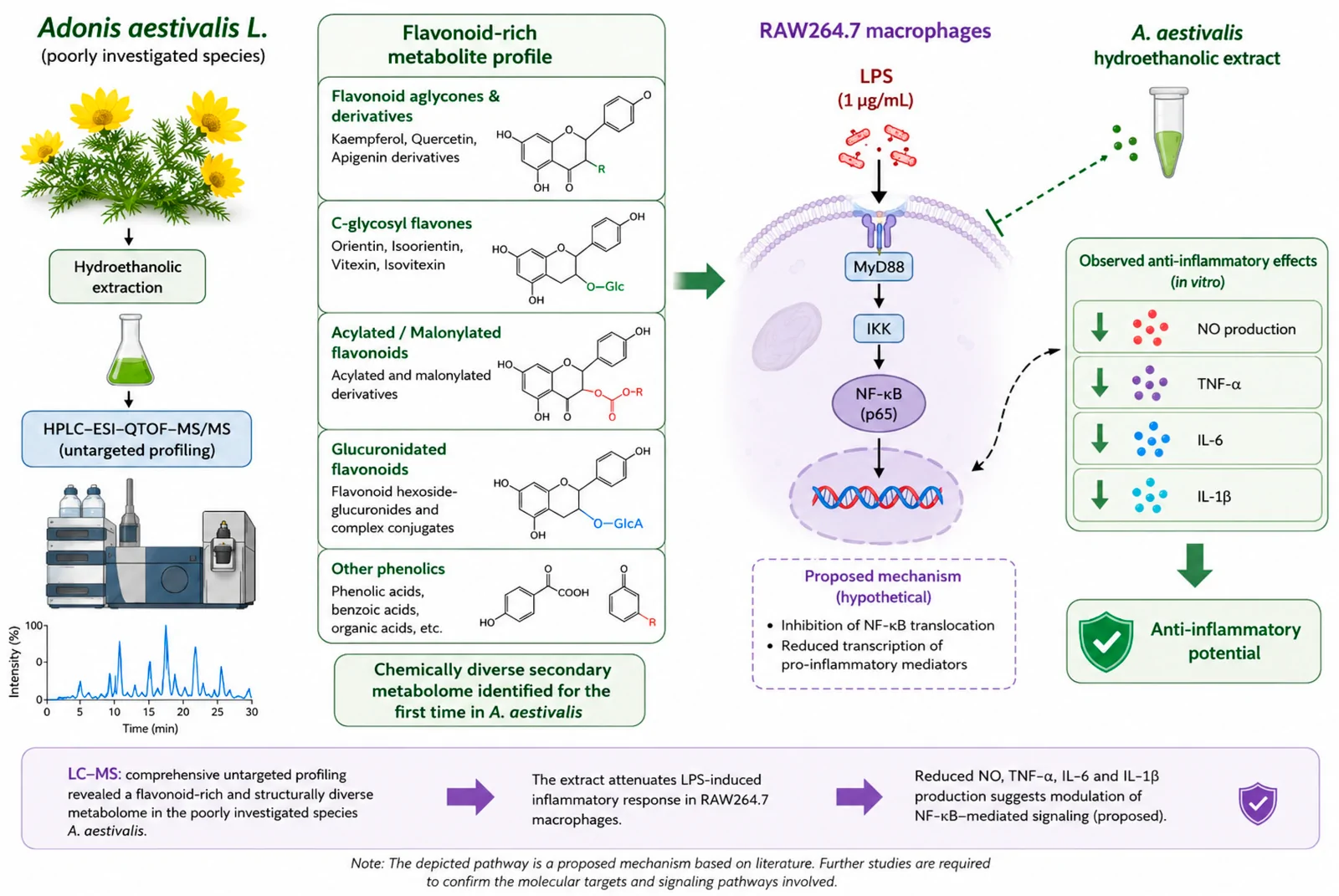
Schematic summary of the study. Untargeted HPLC–ESI–QTOF-MS/MS analysis revealed a flavonoid-rich metabolite profile of the poorly investigated species *Adonis aestivalis*, including numerous tentatively identified flavonoid conjugates. The hydroethanolic extract attenuated LPS-induced inflammatory responses in RAW264.7 macrophages by reducing NO, TNF-α, IL-6 and IL-1β production, suggesting anti-inflammatory potential possibly associated with modulation of NF-κB-related signalling.

The translational potential of the present findings therefore lies primarily in identifying *A. aestivalis* as a candidate for further phytochemical and preclinical investigation. An integrated overview of the phytochemical findings, observed anti-inflammatory effects, and the proposed mechanistic framework is presented in Figure 4. 

Future studies should include quantitative LC–MS analysis using authentic standards, validation of potential chemical markers, assessment of batch-to-batch variability, and bioactivity-guided fractionation followed by testing of isolated compounds and defined combinations. Mechanistic experiments should evaluate iNOS and COX-2 expression and the activation of NF-κB, MAPK, and PPAR-γ pathways. The activity should subsequently be confirmed in primary macrophages and appropriate in vivo inflammation models. Pharmacokinetic, bioavailability, and safety studies will also be required. Because plants of the genus *Adonis* contain cardioactive glycosides, future safety assessment should include their targeted quantification and evaluation of potential cardiac toxicity. Only after these steps could the feasibility of developing a reproducible standardised phytochemical preparation with a defined polyphenolic and safety profile be properly assessed.

## 4. Materials and Methods

### 4.1. Materials

Dimethyl sulfoxide (DMSO, 99.9% purity), lipopolysaccharide (LPS, *Escherichia coli* 0111:B4), foetal bovine serum (FBS), Dulbecco’s modified Eagle’s medium (DMEM), dexamethasone, Griess reagent, and sodium nitrite were purchased from Sigma-Aldrich (Arklow, Ireland). Cell Counting Kit-8 (CCK-8) was obtained from Dojindo (Kumamoto, Japan). Mouse ELISA kits for IL-6, IL-1β, and TNF-α, together with Nunc™ MaxiSorp™ ELISA plates, were purchased from BioLegend (San Diego, CA, USA). Unless otherwise stated, all chemicals were of analytical grade.

### 4.2. The Plant Material

The aerial parts of *Adonis aestivalis* L. were collected during the flowering stage in May 2022 near Uzynagash village, Zhambyl district, Almaty region, Republic of Kazakhstan (43.4690° N, 76.2830° E).

The plant material was taxonomically identified by specialists of the Institute of Botany and Phytointroduction, Committee of Forestry and Wildlife, Ministry of Ecology and Natural Resources of the Republic of Kazakhstan. Plant identification was confirmed by an official identification letter issued by the Institute (Letter No. 01-05/184).

### 4.3. Extraction of Plant Material

The extraction procedure was performed according to the ultrasound-assisted extraction protocol previously described by Orynbekova et al. [28] with minor modifications.

Briefly, 5 g of powdered aerial plant material was extracted with 50 mL of extraction solvent using ultrasonic treatment for 30 min at room temperature. Three solvent systems were investigated: distilled water, absolute ethanol, and ethanol–water (1:1, *v*/*v*). After centrifugation, the supernatants were filtered and evaporated to dryness at 45 °C.

The dried extracts were stored under refrigerated conditions until analysis. Prior to chromatographic analysis, extracts were re-dissolved in the corresponding extraction solvent to obtain a concentration of 10 mg/mL. For biological assays, the hydroethanolic extract was dissolved in dimethyl sulfoxide to prepare a stock solution of 100 mg/mL.

### 4.4. HPLC–ESI–QTOF-MS/MS Fingerprinting of the Extract

Qualitative metabolite profiling was carried out using an Agilent 1200 Series HPLC system coupled to an Agilent G6530B quadrupole time-of-flight mass spectrometer equipped with an electrospray ionisation Jet Stream source (Agilent Technologies, Santa Clara, CA, USA).

Chromatographic separation was carried out on a Zorbax Eclipse Plus RP-18 column (150 × 2.1 mm, 3.5 μm) (Agilent Technologies, Santa Clara, CA, USA) maintained at 20 °C using a flow rate of 0.2 mL/min. The mobile phase consisted of 0.1% formic acid in water (A) and 0.1% formic acid in acetonitrile (B), using a gradient from 98% A to 5% A. MS/MS spectra were acquired in both positive and negative ion modes using collision energies of 10 and 20 V.

Compounds were tentatively identified based on accurate mass measurements, characteristic fragmentation patterns, and comparison with the METLIN database and published literature.

### 4.5. Bioactivity Studies

#### 4.5.1. Cell Culture

RAW264.7 murine macrophages were cultured in high-glucose DMEM supplemented with 10% foetal bovine serum under standard conditions (37 °C, 5% CO_2_), as previously described [28].

#### 4.5.2. Cell Viability Assay

Cell viability was evaluated using the Cell Counting Kit-8 (CCK-8) assay according to the procedure described previously [28].

Briefly, RAW 264.7 cells were seeded into 96-well plates at a density of 1 × 10^5^ cells/well and cultured for 24 h. The cells were then treated with vehicle control (DMSO), dexamethasone (DEX, 5 μg/mL), or the extract of *A. aestivalis* at concentrations of 10, 25, 50, 100, and 200 μg/mL for 24 h. After treatment, the CCK-8 reagent was added according to the manufacturer’s instructions, and absorbance was measured at 450 nm using a BioTek microplate reader. Cell viability was expressed as a percentage of the untreated control. The relative cell viability was calculated using the following equation:

Relative cell viability = [(OD of experimental group)/(OD of control group)] × 100(1)

#### 4.5.3. Determination of NO Production

Nitric oxide production was determined using the Griess assay following the procedure reported previously [28].

RAW 264.7 cells were stimulated with LPS (1 μg/mL) in the presence or absence of the hydroethanolic extract (10, 25, and 50 μg/mL). Dexamethasone (5 μg/mL) served as the positive control. After 24 h of incubation, nitrite levels in the culture supernatants were quantified using Griess reagent according to the manufacturer’s instructions.

#### 4.5.4. Determination of Cytokine Production in Cell Supernatant

The concentrations of IL-6, IL-1β, and TNF-α in cell culture supernatants were determined using commercial ELISA kits according to the manufacturers’ instructions, following the protocol previously described by Orynbekova et al. [28].

RAW264.7 cells were stimulated with LPS (1 μg/mL) and treated with the hydroethanolic extract (10, 25, and 50 μg/mL). Dexamethasone (5 μg/mL) was used as the positive control.

#### 4.5.5. Statistical Analysis

All experiments were performed independently in triplicate, and the results are expressed as mean ± standard deviation (SD). Statistical significance was evaluated using one-way analysis of variance (ANOVA) followed by Dunnett’s multiple comparison test to compare treatment groups with the LPS-treated group. Differences were considered statistically significant at *p* < 0.05.

## 5. Conclusions

This study demonstrated that *Adonis aestivalis* L. exhibits a chemically diverse metabolic profile dominated by tentatively identified flavonoids, including derivatives of kaempferol, quercetin, and apigenin, as well as C-glycosylated flavones, accompanied by phenolic acids, fatty acid derivatives, organic acids, and adonitol. The tentative annotation of glycosylated, acylated, and glucuronidated flavonoid derivatives expands current knowledge of the polyphenolic composition of *A. aestivalis*, while the detection of adonitol supports its possible chemotaxonomic relevance within the genus *Adonis*. However, confirmation using authentic reference standards or complementary structural techniques is required.

The selected 50% ethanolic extract showed no cytotoxicity towards RAW 264.7 cells at concentrations up to 50 μg/mL and produced a statistically significant but moderate reduction in LPS-induced NO production. Variable reductions in the levels of IL-6, IL-1β, and TNF-α were also observed. These findings provide preliminary evidence of the anti-inflammatory potential of the extract. However, because iNOS, NF-κB, MAPK, and other inflammatory targets were not evaluated, the molecular mechanisms underlying these effects remain to be established.

Overall, *A. aestivalis* may be considered a promising source of flavonoid-rich extracts with preliminary anti-inflammatory potential. The present findings provide a foundation for further phytochemical and preclinical investigation rather than direct evidence supporting immediate development of a standardised product. Quantitative LC–MS analysis using authentic standards, validation of suitable chemical markers, bioactivity-guided fractionation, testing of isolated compounds, mechanistic studies, safety assessment—including targeted evaluation of cardioactive glycosides—and in vivo validation are required before the feasibility of developing a standardised phytochemical preparation with a defined polyphenolic profile can be established.

## Figures and Tables

**Table 1:** The tentatively identified metabolites of *Adonis aestivalis* in the HPLC-ESI-QTOF-MS/MS analysis (Diff—error of measurement, DBE—double bond and rings number).

No	Ion	Retention Time [min]	Compounds	Neutral Formula	Theoretical Mass	Precursor Ion *m*/*z*	Diff [ppm]	DBE	Products Ions [*m*/*z*]
Flavonoids									
1	[M–H]^−^	18.025	Saponarin	C_27_H_30_O_15_	593.1512	593.1506	1.0	13	473.1086 431.0984 311.0562
2	[M–H]^−^	18.1	Kaempferol-*O*-diglucoside	C_27_H_30_O_16_	609.1461	609.1456	0.83	13	447.0949
3	[M–H]^−^	18.108	Orientin glucoside	C_27_H_30_O_16_	609.1461	609.1457	0.67	13	519.1196 489.1076 447.0961 369.0632 357.0632 327.0528 297.0437 285.0390
4	[M–H]^−^	18.458	Quercetin hexoside glucuronide	C_27_H_28_O_18_	639.1203	639.1203	−0.02	14	463.0874 301.0349
5	[M–H]^−^	19.442	Isoorientin	C_21_H_20_O_11_	447.0933	447.0933	0.03	12	369.0560 357.0506 327.0421 297.0342 285.0337
6	[M–H]^−^	19.596	Vitexin	C_21_H_20_O_10_	431.0984	431.0988	−0.99	12	341.0667 311.0567 283.0621
7	[M–H]^−^	19.842	Acetylated luteolin C-hexoside (tentative)	C_23_H_22_O_12_	489.1038	489.1035	0.71	13	429.0832 393.0619 357.0616 327.0502 297.0366 285.0406
8	[M–H]^−^	19.892	Esterified quercetin conjugate	C_37_H_36_O_22_	831.1625	831.1615	1.26	20	669.1101 639.1200 463.0872 431.0979 367.0648 301.0330 191.0343
9	[M–H]^−^	19.9	Isovitexin	C_21_H_20_O_10_	431.0984	431.0984	−0.07	12	341.0667 311.0567 283.0621 161.0440
10	[M–H]^−^	20.092	Malonylated luteolin C-hexoside (tentative)	C_24_H_22_O_14_	533.0937	533.0961	−4.53	14	489.1057 429.0843 357.0624 327.0527 297.0417 285.0403
11	[M–H]^−^	20.309	Orientin	C_21_H_20_O_11_	447.0933	447.0922	2.42	12	357.0611 327.0509 297.0395 285.0391
12	[M–H]^−^	20.576	Apigenin-O-(malonyl)-hexoside	C_24_H_22_O_13_	517.0988	517.0953	6.69	14	473.1026 413.0796 341.0590 311.0486 283.0537 269.0370
13	[M–H]^−^	21.443	Kaempferol	C_15_H_10_O_6_	285.0405	285.0406	−0.48	11	175.0426
Phenolic acids									
14	[M–H]^−^	20.91	Dicaffeoylquinic acid	C_25_H_24_O_12_	515.1195	515.1191	0.77	14	473.1066 353.0862 311.0527 179.0337 135.0442
15	[M–H]^−^	17.407	Chlorogenic acid	C_16_H_18_O_9_	353.0878	353.089	−3.37	8	191.0562 161.0254
Organic acids									
16	[M–H]^−^	27.131	Hydroxypalmitic acid	C_16_H_32_O_3_	271.2279	271.2271	2.82	1	225.2221
17	[M–H]^−^	25.368	Conjugated linoleic acid	C_18_H_32_O_2_	279.2330	279.2339	−3.38	3	-
18	[M–H]^−^	22.6	Dihydroxy-octadecanoic acid	C_18_H_32_O_4_	311.2228	311.2234	−1.98	3	293.2138 265.1821 235.1718 223.1713 201.1149
19	[M–H]^−^	2.01	Malic acid	C_4_H_6_O_5_	133.0142	133.0153	−7.86	2	115.0022
20	[M–H]^−^	2.099	Citric acid	C_6_H_8_O_7_	191.0197	191.0198	−0.38	3	173.0096 111.0092 87.0098
Organic alcohols									
21	[M–H]^−^	2.065	Adonitol	C_5_H_12_O_5_	151.0612	151.0604	5.24	0	119.0336 101.0230 89.0234 71.0130

## Data Availability

Data are contained within the article or Supplementary Materials.

## Supplementary Materials

The following supporting information can be downloaded at: https://www.mdpi.com/article/10.3390/molecules31162858/s1, Figure S1. The total mass chromatogram of *A. aestivalis* recorded in the negative and positive ion modes of the obtained extracts using 50% EtOH, 96% EtOH and HOH; Table S1: The MS/MS spectra recorded for the majority of the tentatively identified molecules from Table 1.
