# Supplementary material for: Flavonoid-Rich Metabolite Profiling and Anti-Inflammatory Activity of Adonis aestivalis L
Source: Molecules. 2026 Aug 16;31(16):2858. doi: 10.3390/molecules31162858 (PMC13515396; doi:10.3390/molecules31162858)

Figure S1. The total mass chromatogram of *A. aestivalis* recorded in the negative and positive ion modes of the obtained extracts using 50% EtOH, 96% EtOH and HOH.

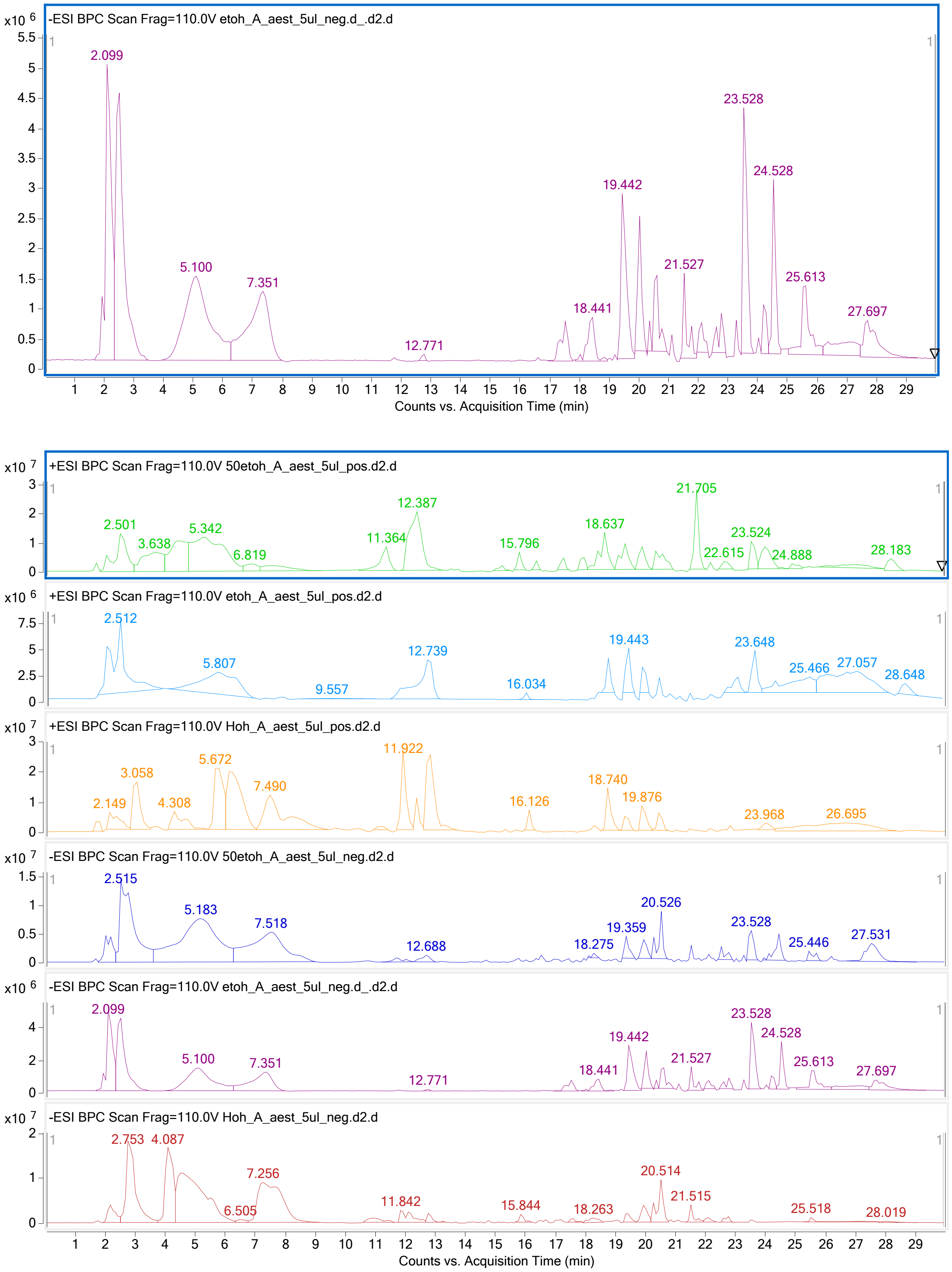

Supplement: Supplementary file 1 [file molecules-31-02858-s001.zip › figure S1.pdf]
