# Supplementary material for: Flavonoid-Rich Metabolite Profiling and Anti-Inflammatory Activity of Adonis aestivalis L
Source: Molecules. 2026 Aug 16;31(16):2858. doi: 10.3390/molecules31162858 (PMC13515396; doi:10.3390/molecules31162858)
Supplement: Supplementary file 1 [file molecules-31-02858-s001.zip › table s1.pdf]

SUPPLEMENTARY FILE

Table S1. The MS/MS spectra recorded for the majority of the tentatively identified molecules from Table 1.

No	Retention time [min]	Proposed compound	MS/MS spectrum
1.	2.065	Adonitol	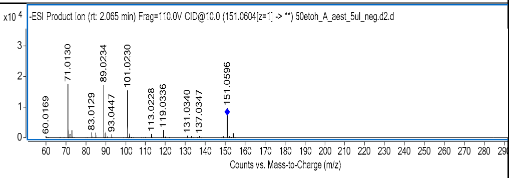
2.	2.565	Malic acid	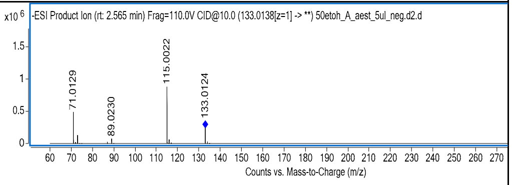
3.	2.982	Citric acid	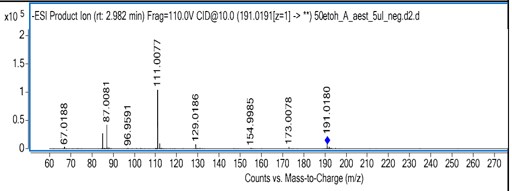
4.	17.407	Chlorogenic acid	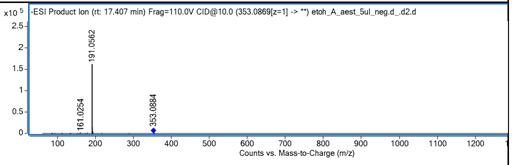
5.	18.025	Saponarin	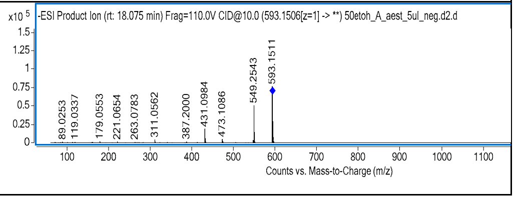

6.	18.1	Kaempferol O-diglucoside	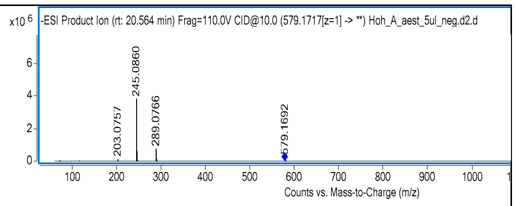
7.	18.108	Orientin glucoside	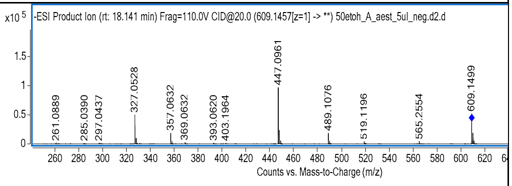
8.	18.458	Quercetin hexoside glucuronide	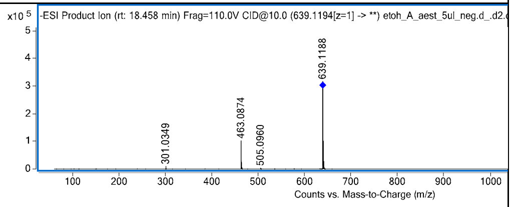
9.	19.442	Isorientin	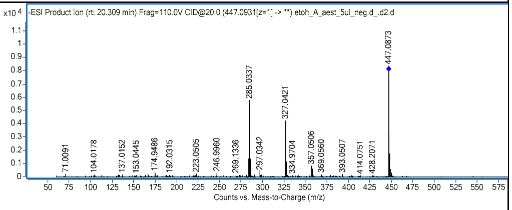
10.	19.596	Vitexin	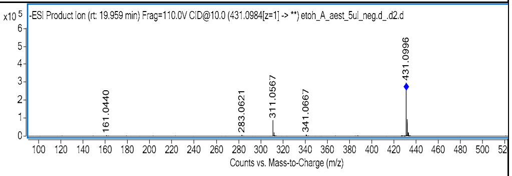
11.	19.842	Kaempferol acetylgalactoside	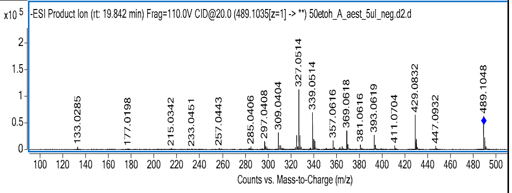
12.	19.892	Estrified Quercetin hexoside -glucuronide	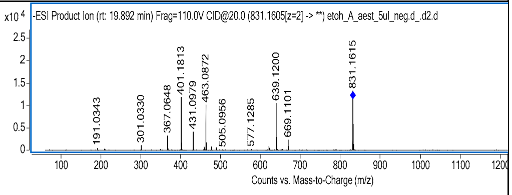

13.	19.9	Isovitexin	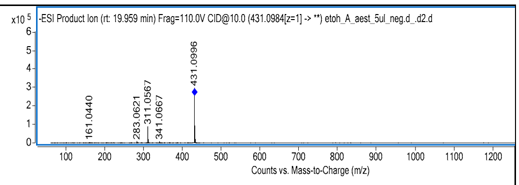
14.	20.092	Kaempferol malonylglucoside	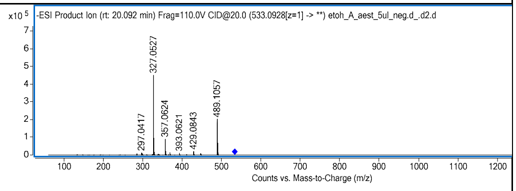
15.	20.309	Orientin	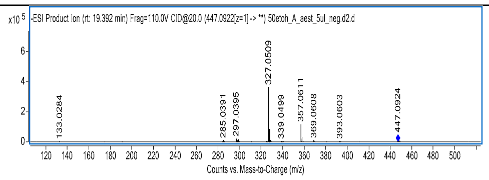
16.	20.576	Apigenin-O-(malonyl)-hexoside	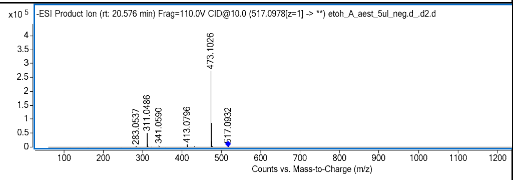
17.	20.91	Dicaffeoylquinic acid	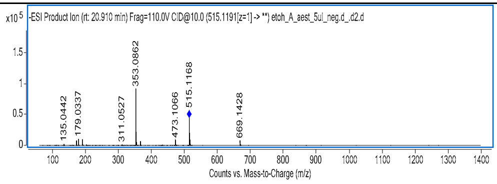
18.	21.443	Kaempferol	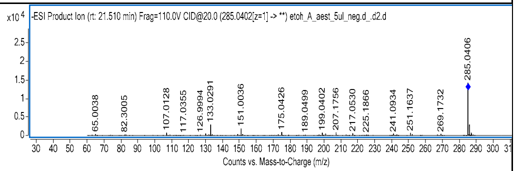
19.	22.52	Embelin	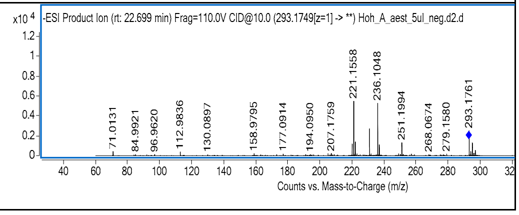

<table>
<tr><td>20.</td><td>22.6</td><td>dihydroxy-octadecanoic acid</td><td>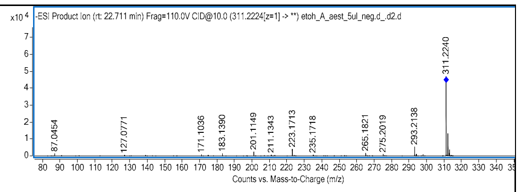
</td></tr>
<tr><td>21.</td><td>25.368</td><td>Conjugated linoleic acid</td><td>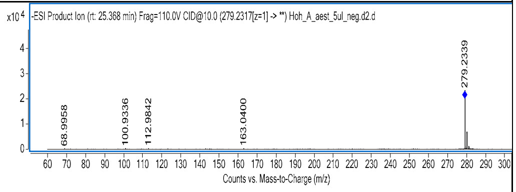
</td></tr>
<tr><td>22.</td><td>27.131</td><td>Hydroxypalmitic acid</td><td>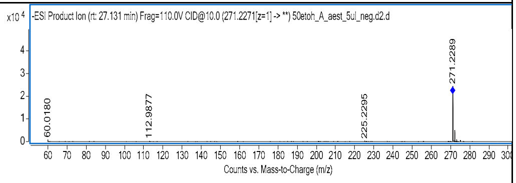
</td></tr>
</table>
